# Fungating Breast Carcinoma Complicated by Carbapenem-Resistant Pseudomonas aeruginosa Infection in a Patient With Severe Blood-Injection-Injury Phobia: A Case Report

**DOI:** 10.7759/cureus.103978

**Published:** 2026-02-20

**Authors:** Amia Mourad, Nicholas Lorenz, Krishna Patel, Ishan Patel, Mithun Pattathan

**Affiliations:** 1 Medicine, Lake Erie College of Osteopathic Medicine, Bradenton, USA; 2 Internal Medicine, St. Vincent's Medical Center, Jacksonville, USA

**Keywords:** blood phobia, delayed presentation, fungating breast mass, sepsis, severe anemia, surgical debridement

## Abstract

Fungating breast tumors represent advanced breast cancer and are frequently complicated by secondary infection. While bacterial colonization is common, infection with carbapenem-resistant *Pseudomonas aeruginosa* (CR-PSA) in breast wounds is rare and poses significant therapeutic challenges. We report the case of a 60-year-old woman who presented with a six-month history of a progressively enlarging fungating right breast mass, with delayed evaluation due to severe blood-injection-injury (BII) phobia. On admission, she was septic and profoundly anemic. Imaging demonstrated a large exophytic breast mass with bilateral axillary lymphadenopathy, and biopsy confirmed grade 3 invasive mammary carcinoma. Wound cultures grew CR-PSA, necessitating intravenous antibiotics, surgical debridement, and targeted antimicrobial therapy. Her clinical course required blood transfusions and multiple specialities consulted prior to stabilization and discharge for outpatient oncologic care. This case highlights the potential for fungating breast tumors to harbor multidrug-resistant organisms and illustrates how psychological barriers to care can contribute to advanced disease and complex clinical presentations.

## Introduction

Fungating breast tumors represent a severe and advanced stage of breast cancer in which malignant tissue infiltrates and outgrows the skin, leading to ulceration, necrosis, and malodorous drainage [1]. Malignant fungating wounds occur in approximately 5%-14% of patients with advanced cancer in the United States, and fungating breast tumors represent a subset of these lesions and therefore account for a smaller proportion of all breast cancer presentations [1]. These lesions are consistently associated with profound physical, psychological, and social burdens. The breakdown of the skin barrier, impaired lymphatic drainage, exposure of necrotic tissue, and chronic exudate create an ideal environment for microbial colonization [1]. While colonization is common, true infection, particularly with virulent or multidrug resistant organisms, can substantially worsen outcomes by accelerating tissue destruction, triggering systemic inflammatory responses, and complicating wound care [1].

The microbiology of fungating breast wounds typically includes a mixture of skin flora and anaerobic organisms such as *Staphylococcus aureus*, *Streptococcus* species, mixed anaerobes, and Enterobacteriaceae [2]. Among gram-negative bacteria, *Pseudomonas aeruginosa* is frequently present in moist, necrotic, or long-standing lesions. Carbapenem-resistant *P. aeruginosa* (CR-PSA) represents a particularly challenging pathogen due to its intrinsic resistance mechanisms, its capacity to acquire additional resistance determinants, and its limited susceptibility to available antimicrobial agents [3]. CR-PSA is typically associated with healthcare exposure, with major risk factors including prior carbapenem use, medical devices, ICU admission, mechanical ventilation, and prior antibiotic therapy [4]. Its occurrence in a patient without prior healthcare exposure is clinically significant.

Delays in seeking medical care are frequently associated with the development of advanced, fungating breast tumors [5]. Barriers to early detection reported in the literature include demographic and socioeconomic factors such as being single, post-menopausal, and having low income, as well as limited access to healthcare services and lack of insurance [6]. Knowledge and awareness related barriers include lack of understanding or practice of breast self examination [6]. Additional contributors include fear of diagnosis, mistrust of medical systems, cultural beliefs, stigma, prior negative healthcare experiences, and psychiatric conditions such as blood-injection-injury (BII) phobias [6]. BII phobia, a specific phobia characterized by intense fear of seeing blood, bodily injury, or medical procedures, can lead to profound avoidance of healthcare encounters [7]. This avoidance may delay routine screening, evaluation of early breast symptoms, and management of progressively worsening lesions. The resulting diagnostic delay not only increases the likelihood of advanced tumor stage at presentation but also raises the risk of secondary infection, anemia, and cancer related complications.

In this report, we present a rare case of fungating breast carcinoma complicated by CR-PSA infection in a woman whose long-standing BII phobia delayed care, demonstrating how multidrug resistance and psychological barriers can converge to worsen clinical outcomes.

## Case presentation

A 60-year-old woman with no documented past medical history presented to the emergency department with a six-month history of a progressively enlarging wound beneath her right breast. She reported that the lesion began as two small skin abnormalities that steadily increased in size, eventually forming a large open ulcer with drainage and odor. She endorsed intermittent fevers, chills, worsening fatigue, and progressive shortness of breath with minimal exertion. Over the preceding weeks, she also noticed swelling of her right arm and hand. The patient attributed her delay in seeking medical care to a long-standing and severe BII phobia, which led her to avoid medical evaluation despite progressive deterioration.

**Figure 1:**
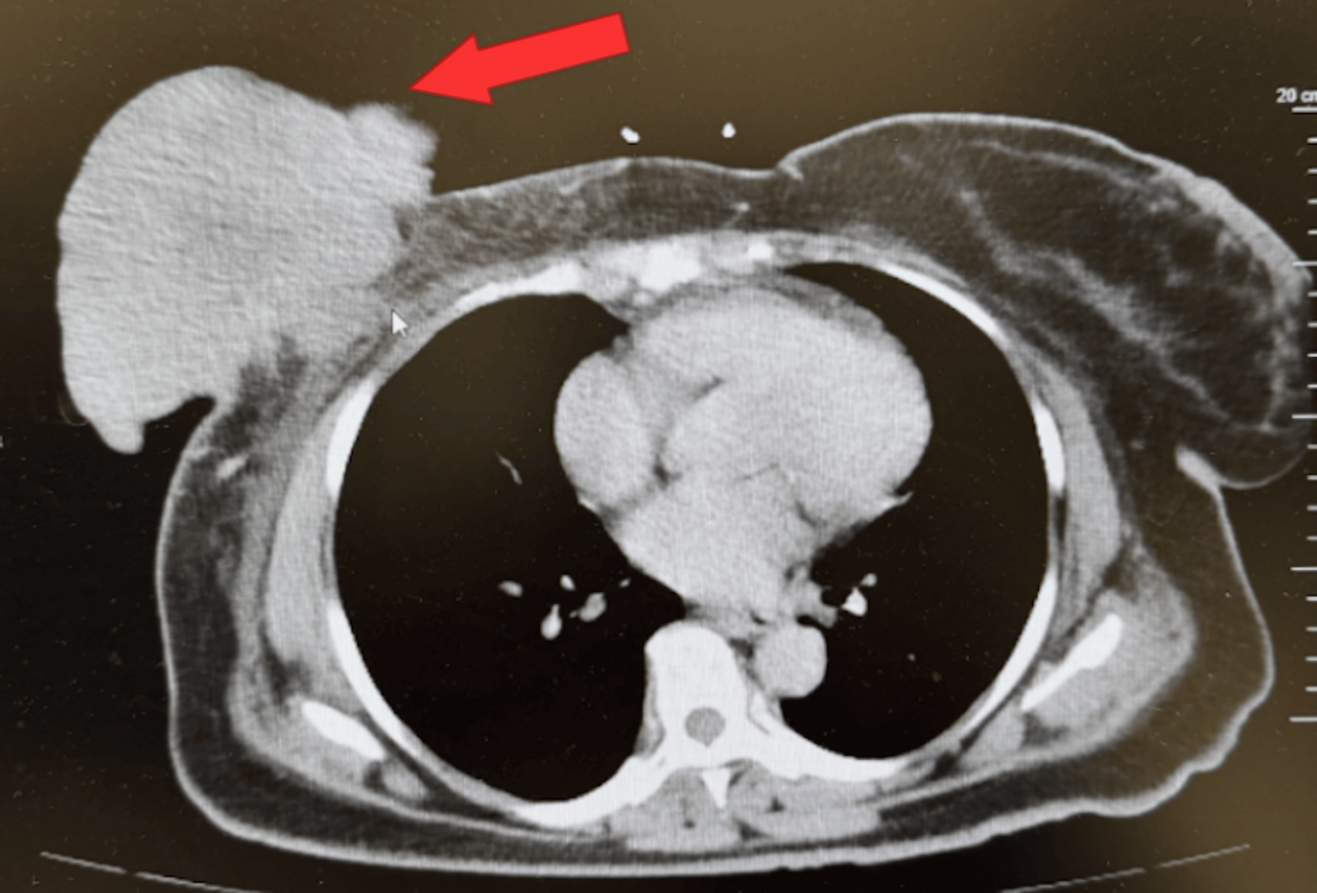
Contrast enhanced CT scan of the chest, abdomen, and pelvis demonstrating a large exophytic right breast mass (red arrow).

On arrival, she was febrile, tachycardic, and markedly pale (Table 1). Laboratory evaluation on admission revealed profound anemia, leukocytosis, and elevated lactate, consistent with systemic compromise (Table 1). The patient’s severe anemia was multifactorial. The contributing factors included chronic inflammation from advanced malignancy, iron deficiency, and likely chronic low-grade blood loss from friable, ulcerated tumor tissue. There was no evidence of brisk or active hemorrhage from the wound. She was diagnosed with severe anemia, sepsis, and a right breast wound concerning for malignancy. Blood cultures were obtained and the patient was started on intravenous fluids and empiric broad spectrum intravenous antimicrobial therapy with cefepime and vancomycin. CT imaging of the chest, abdomen, and pelvis revealed a large exophytic right breast mass with additional breast lesions, associated skin thickening, and bilateral axillary lymphadenopathy (Figure 1). The radiologic appearance was strongly suggestive of primary breast malignancy with metastatic nodal involvement. Physical examination demonstrated a large fungating mass on the inferior right breast with a necrotic base and purulent drainage. Management involved coordination among multiple specialties, including surgery, infectious disease, hematology and oncology, to address both the malignant and infectious components of her presentation.

**Table 1:** Admission Laboratory Values and Vital Signs

Parameter	Result	Reference Range
Temperature	38.1°C	36.1-37.2°C
Heart Rate	112 bpm	60-100 bpm
Blood pressure	98/60 mmHg	90/60-120/80 mmHg
Hemoglobin	3.5 g/dL	12-16 g/dL
Hematocrit	13.4 %	36-46%
White blood cells	12.2×10³/µL	4.0-11×10³/µL
Platelets	523×10³/µL	150-400×10³/µL
Lactic acid	3.4 mmol/L	0.5-2.2 mmol/L

The patient was admitted to a progressive care unit and received four units of packed red blood cells, resulting in improvement of hemoglobin to 7.8 g/dL over the following days. After clinical stabilization and improvement in her systemic inflammatory response, iron studies demonstrated iron-deficiency concurrent with anemia of chronic disease, and intravenous ferric gluconate along with vitamin B12 supplementation was initiated. Biopsy of the mass revealed grade 3 invasive mammary carcinoma, with tumor cells staining positive for GATA-3 and CK7. She was found to have associated right upper extremity lymphedema and superficial thrombosis of the cephalic vein, attributed to tumor burden and inflammatory changes.

Despite initial improvement in fever, the patient continued to have intermittent elevations in temperature. Surgical evaluation recommended operative debridement due to extensive devitalized tissue. In the operating room, a large necrotic tumor segment measuring approximately 15 cm was debrided, removing a 10×14×8 cm area of nonviable tissue. Pathology from debridement confirmed invasive carcinoma with abundant necroinflammatory debris. Superficial wound cultures subsequently grew CR-PSA that remained susceptible to ciprofloxacin (minimum inhibitory concentration (MIC), 0.5 µg/mL). Deep surgical cultures were not obtained due to extensive tumor necrosis, difficulty distinguishing viable from devitalized tissue, and the risk of bleeding. 

Based on the susceptibility results, the infectious disease specialists recommended targeted oral therapy with ciprofloxacin 500 mg twice daily and trimethoprim-sulfamethoxazole DS twice daily for 10 days upon discharge. The patient received intravenous cefepime and vancomycin for a total of 10 days prior to transition to oral step-down therapy with ciprofloxacin and trimethoprim-sulfamethoxazole at discharge. Trimethoprim-sulfamethoxazole was added to broaden antimicrobial coverage and reduce the risk of resistance development during oral therapy in the setting of a complex malignant wound. Although fluoroquinolone resistance and clinical failure are concerns in complex *Pseudomonas* infections, this patient demonstrated clinical improvement following surgical source control and intravenous therapy, with persistently negative blood cultures and no evidence of bacteremia. Given confirmed ciprofloxacin susceptibility, limited alternative oral options, and the need for outpatient management, infectious disease specialists recommended oral ciprofloxacin with adjunct trimethoprim-sulfamethoxazole as step-down therapy.

The patient’s hemoglobin stabilized near 8 g/dL after repeated transfusions and her vital signs normalized. She was discharged with plans for outpatient port placement, initiation of systemic chemotherapy, and ongoing wound care management.

## Discussion

The clinical course in this case was shaped less by the presence of a fungating breast tumor itself and more by the downstream consequences of delayed presentation, including physiologic collapse and infection with a highly resistant organism. Advanced malignancy may initially manifest as physiologic instability rather than a purely oncologic problem, requiring medical stabilization before definitive cancer therapy can proceed. Recognizing this possibility is critical and shows the importance of early collaboration among surgery, infectious disease, and oncology teams in patients with advanced malignant wounds.

Fungating breast tumors are frequently colonized by bacteria; however, published studies report that most associated infections are superficial, polymicrobial, and caused by antibiotic-susceptible organisms, with systemic infection occurring infrequently [2]. In a retrospective review of 64 symptomatic cutaneous metastases, pathogenic bacteria were isolated from roughly half of lesions, with *S. aureus* and *P. aeruginosa* being the most frequent organisms identified [8]. Patients treated with oral antibiotics demonstrated better symptomatic improvement, suggesting that standard antimicrobial therapy is often effective for symptomatic lesions in that setting [8]. In contrast, our patient’s wound culture grew CR-PSA, a multidrug-resistant phenotype not characterized in that cohort, and the infection progressed to systemic sepsis requiring targeted antimicrobial therapy and surgical debridement. This divergence from expected microbiologic patterns shows the importance of early culture acquisition and resistance profiling in malignant wounds, particularly in the setting of extensive necrosis and delayed presentation.

Carbapenem resistance in gram-negative bacteria can arise through multiple mechanisms. These include production of carbapenem-hydrolyzing enzymes (carbapenemases), loss or mutation of outer membrane porins, overexpression of multidrug efflux pumps, and derepression of chromosomal β-lactamases such as AmpC [3,9]. In *P. aeruginosa*, resistance most often develops through porin loss and efflux pump overexpression, with carbapenemase production being less frequently the dominant mechanism [3]. Although the patient’s CR-PSA was resistant to carbapenems, it remained susceptible to ciprofloxacin. This finding indicates a non-carbapenemase-mediated mechanism, as carbapenemase-producing strains often exhibit broader multidrug resistance that limits oral treatment options [3,9]. This is not to say that carbapenemases are uncommon in other organisms. For example, Enterobacteriaceae often achieve carbapenem resistance through plasmid-mediated carbapenemases such as *Klebsiella pneumoniae* carbapenemase (KPC), New Delhi metallo-β-lactamase (NDM), Verona integron-encoded metallo-β-lactamase (VIM), and oxacillinase (OXA) types [3]. This is sometimes combined with porin alterations, which can confer high-level resistance and facilitate horizontal transfer [3,9]. Recognizing these mechanistic distinctions is clinically important, as they shape empiric antibiotic selection, and guide targeted susceptibility testing.

Profound anemia further compounded the patient’s vulnerability, likely worsening both her septic physiology and tolerance of infection and surgical intervention. While anemia is a recognized complication of advanced cancer, the degree observed in this patient (hemoglobin 3.5 g/dL) far exceeds levels typically reported in breast cancer-associated anemia, which is more commonly mild to moderate (8-10 g/dL) [10]. Severe anemia impairs oxygen delivery to tissues, diminishes immune function, and reduces physiologic reserve, amplifying septic responses rather than acting as a coincidental finding [11]. Correction of the anemia was essential not only for hemodynamic stabilization but also for enabling infection control and subsequent cancer directed therapy. This highlights the critical role of physiologic optimization in patients with advanced malignant wounds.

Delayed presentation was central to the severity of this case, and the underlying cause, severe BII phobia, introduces an important dimension of help‑seeking avoidance not commonly addressed in oncologic literature. In a qualitative synthesis of international studies on delay in cancer presentation, Smith and colleagues found that fear of consultation was a pervasive theme across cancer types. These manifested as fear of embarrassment, fear of cancer pain and suffering, or fear of invasive procedures, all of which contributed to prolonged help‑seeking intervals in more than 700 patients studied [12]. While Smith et al. did not examine BII phobia, the fear-related patterns they describe parallel our patient’s extreme avoidance of medical evaluation, which resulted in unchecked tumor growth and disease progression. Importantly, the synthesis highlighted that symptom recognition and interpretation are key determinants of timely presentation [12]. Also, the presence of encouragement from friends and family can facilitate earlier care, while absence of such support may prolong delay [12]. Unlike many patients in the reviewed studies who experienced more typical fears (e.g., fear of diagnosis or embarrassment), our patient’s phobia was specific and absolute, resulting in near complete avoidance of health care until life‑threatening complications developed. Recognition of such intense psychological barriers is critical, as they are potentially modifiable through targeted screening, mental health referral, or alternative diagnostic pathways that address both symptom interpretation and the emotional hurdles that prevent people from seeking timely care.

## Conclusions

This case highlights several important clinical lessons. Fungating breast tumors can serve as active infectious sources rather than purely palliative lesions. In patients with advanced malignant wounds, particularly those with extensive necrosis, systemic infection, or delayed presentation, early culture acquisition and antimicrobial resistance testing should be prioritized. Severe anemia can both signal and exacerbate sepsis, emphasizing the need for prompt physiologic optimization. Additionally, psychological barriers, including BII phobia, may delay cancer diagnosis and should be considered in high-risk patients. Finally, complex presentations such as this show the importance of early multidisciplinary coordination, involving surgery, infectious disease, hematology, and oncology, to optimize patient outcomes in advanced malignancy complicated by multidrug resistant infection.
